# Melioidosis with Portal Vein Thrombosis

**DOI:** 10.1155/2017/2891510

**Published:** 2017-04-03

**Authors:** Thanyaporn Direksunthorn

**Affiliations:** School of Medicine, Walailak University, Nakhon Si Thammarat, Thailand

## Abstract

Melioidosis, caused by* Burkholderia pseudomallei*, is a common infectious disease in tropical regions. The author reports a case of melioidosis with a rare manifestation of portal vein thrombosis and cavernous transformation of the portal vein. Melioidosis should be considered a differential diagnosis in patients with underlying risk factors who present with multiple liver abscesses; moreover, portal vein thrombosis can be a potential complication. Computed tomography is the modality of choice to demonstrate venous thrombosis in various organs.

## 1. Introduction

Melioidosis, caused by* Burkholderia pseudomallei*, is a common infectious disease in tropical and subtropical regions and is especially endemic in Southeast Asia [1]. Since 1997, the number of melioidosis cases diagnosed in Thailand has been increasing [2], with approximately 1,000 deaths per year [3]. The lung is the most commonly affected organ [4, 5], and the spleen is the most commonly affected intra-abdominal organ, followed by the liver and the kidney [6]. Computed tomography (CT) can reveal the radiologic characteristics of melioid abscesses such as the “necklace sign” or the “honeycomb sign” [7]. The author reports here a case of melioidosis with a rare manifestation of portal vein thrombosis.

## 2. Case Presentation

A written informed consent was obtained from the patient for publication of this case report and all accompanying images.

An otherwise healthy 54-year-old Thai farmer, a resident in Nakhon Si Thammarat, visited the emergency department because of persistent fever with right upper abdominal pain for 1 month. He had no medical or surgical history of note, in particular, and no known history of diabetes or immunosuppression. He sought local care and was given oral antibiotics and antipyretics, but his condition had not improved.

On arrival, he was febrile, icteric, and dehydrated. His body temperature was 38.9°C, blood pressure was 100/70 mmHg, respiratory rate was 30/min, and heart rate was 110/min. The liver was mildly enlarged. No cardiovascular or respiratory abnormality was found on physical examination.

Laboratory analysis showed the following: leukocytosis (total leukocyte count, 16,700 cells/mm^3^; segmented neutrophils, 80%); fasting blood sugar, 202 mg/dl; glycosylated hemoglobin (HbA1c), 12.8%; total bilirubin, 4.74 mg/dl; direct bilirubin, 3.49 mg/dl; alkaline phosphatase, 553 U/L; serum glutamic-oxaloacetic transaminase (SGOT), 27 U/L; and serum glutamic-pyruvic transaminase (SGPT), 39 U/L. Serum total protein and albumin levels were 7.2 and 3.6 g/dl, respectively. Results of coagulation tests of prothrombin time (PT) and international normalized ratio (INR) were within normal limits. Blood culture results showed* Burkholderia pseudomallei*.

**Figure 1:**
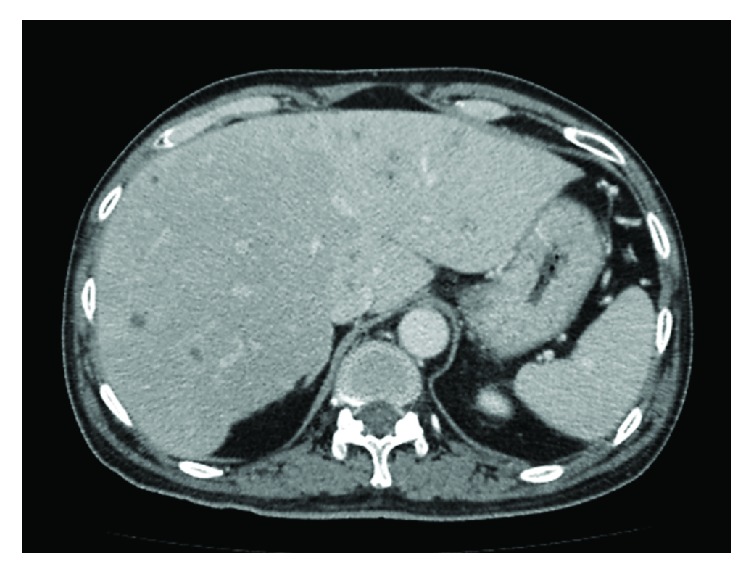
Axial computed tomography (CT) scan (portal venous phase) shows multiple small abscesses throughout both lobes of the liver. Transient hepatic attenuation differences (THAD) due to left portal vein thrombosis were well visualized. Note the normal-appearing spleen.

**Figure 2:**
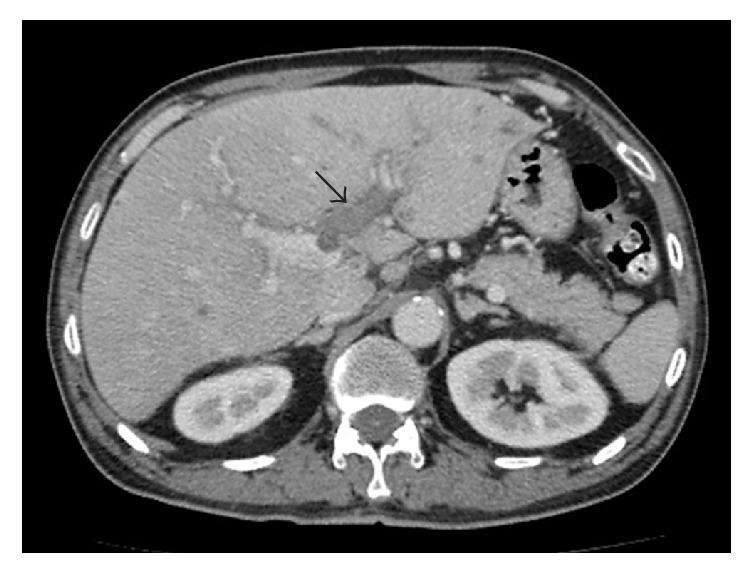
Axial computed tomography (CT) scan (portal venous phase) shows thrombosed left portal vein (arrow). Multiple small periportal vessels, which represent dilated collateral veins, which is the so-called cavernous transformation. Left portal vein thrombosis caused transient hepatic attenuation differences (THAD) between the left and right lobes of the liver.

**Figure 3:**
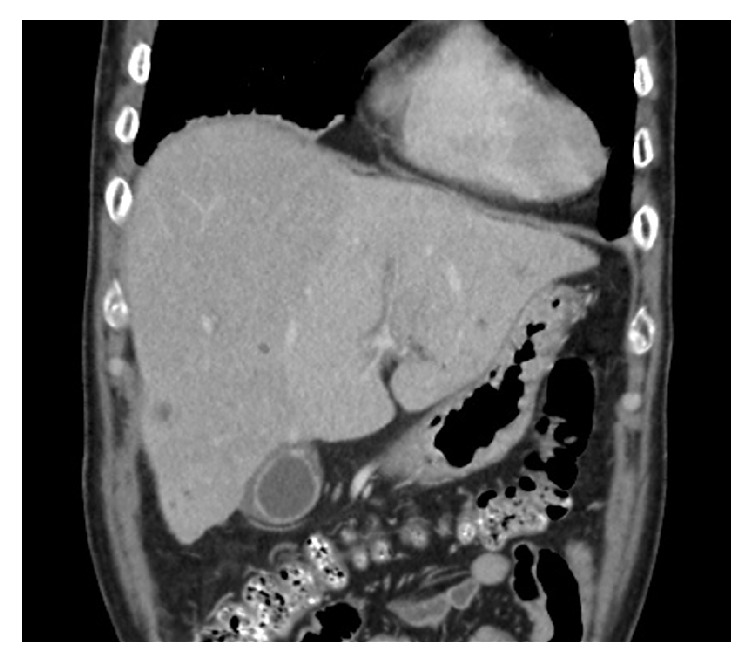
Coronal computed tomography (CT) scan (portal venous phase) shows gallbladder wall congestion and the periportal halo is visualized in the left lobe of the liver.

His CT study showed multiple small liver abscesses, left portal vein thrombosis, and cavernous transformation of the portal vein (Figures 1 and 2). His pancreas and spleen appeared normal. Gallbladder wall congestion and periportal halo were also noted (Figure 3). The chest radiograph showed no pulmonary infiltration or nodule.

The treatment comprised an initial 2-week acute-phase intravenous therapy consisting of ceftazidime and metronidazole, followed by oral amoxicillin-clavulanic acid for 12 weeks, in addition to anticoagulants (daily subcutaneous injections of low molecular weight heparin). Percutaneous abscess drainage was not performed because of small abscesses and the patient's positive response to antibiotics. Improvements in the patient's abdominal pain and blood test results were obtained. A follow-up CT scan was done after 8 weeks of treatment, showing a completely recanalized left portal vein. At the 10-month follow-up after completion of the antibiotic course, the patient had totally recovered and no relapse had been documented. The patient was advised to avoid direct contact with soil or environmental water, to protect wounds from soil and water, and to avoid walking barefoot.

## 3. Discussion

*Burkholderia pseudomallei* (formerly,* Pseudomonas pseudomallei*) is a bipolar-staining, Gram-negative aerobic bacillus and is found in tropical regions. Melioidosis occurs more frequently in patients with underlying diseases, such as diabetes, chronic renal failure, alcoholism, malignancy, and hematological diseases, including immunosuppression. This patient was first diagnosed with diabetes in this visit and it was noted that he worked as a farmer who sometimes walked barefoot on his cultivated land.

The author reports a case of melioidosis with a rare manifestation of portal vein thrombosis and cavernous transformation diagnosed based on CT findings. There are a few reported cases of melioidosis-associated venous thrombosis. Saïdani et al. [8] reported a case of disseminated melioidosis with pulmonary and liver abscesses, in addition to splenic vein thrombosis. Splenic vein thrombosis is also described in cases of melioidosis associated with pancreatic lesions [9]. Niyasom et al. [10] reported a 42-year-old Thai man suffering from septicemic melioidosis with dural sinus thrombosis. The thrombotic events were possibly caused by an inflammatory response to systemic* B. pseudomallei* infection, leading to depletion of the natural endothelial modulators protein C, protein S, and antithrombin [10, 11]. LaRosa et al. [11] studied the correlation between inflammation and coagulation in melioidosis sepsis, revealing that consumption of endothelial modulators was a key feature of this process. Protein C was the modulator most profoundly affected, followed by antithrombin and protein S.

Cavernous transformation of the portal vein is a consequence of portal vein thrombosis and is the replacement of the normal single channel portal vein with numerous tortuous venous channels. Multiphase CT can be used to confirm the diagnosis as it demonstrates numerous vascular structures in the region of the portal vein, which are enhanced during the portal venous phase.

Saverymuttu et al. [12] reported that portal hypertension, not hypoalbuminemia, was the dominant factor causing gallbladder wall thickening in chronic liver disease. The CT image (Figure 3) showed gallbladder wall congestion (so-called congestive cholecystopathy) and a periportal halo. Periportal halos are defined as circumferential zones of low attenuation around the peripheral or subsegmental portal venous branches on contrast-enhanced CT. These halos represent fluid or dilated lymphatics in the loose areolar zone around the portal triad. This sign is nonspecific and can be seen in various conditions.

Transient hepatic attenuation differences (THAD) are localized mismatches in hepatic arterial and portal venous blood supply; often there is a relative increase in hepatic arterial supply, thereby giving a higher attenuation to the affected region. In this case the CT demonstrated higher attenuation in the left lobe of the liver than in the right lobe owing to left portal vein thrombosis.

Various causative organisms have been reported as causing portal vein thrombosis (Table 1).

## 4. Conclusion

Melioidosis should be considered as a differential diagnosis in patients with underlying risk factors who present with multiple liver abscesses; portal vein thrombosis can be a potential complication. Contrast-enhanced CT scan is the modality of choice to identify venous thrombosis in various organs.

## Figures and Tables

**Table 1:** List of causative organisms that have been reported as causing portal vein thrombosis (PVT).

Causative organisms	Presentation
Cytomegalovirus (CMV) [13–15]	Acute CMV-related hepatitis with PVT
*Mycobacterial tuberculosis* [16]	Peritoneal tuberculosis with PVT
Vancomycin resistant *Staphylococcus aureus* (VRSA) [17]	VRSA mandibular osteomyelitis and PVT with hepatic abscess
Group C streptococcus [18]	Sigmoid diverticulitis with PVT
*Escherichia coli *[19]	Sigmoid diverticulitis with PVT
*Fusobacterium necrophorum *[20]	Acute attack on chronic pancreatitis with PVT and multiple hepatic abscesses
